# Effectiveness of synthetic versus autologous bone grafts in foot and ankle surgery: a systematic review and meta-analysis

**DOI:** 10.1186/s12891-024-07676-8

**Published:** 2024-07-13

**Authors:** Amir Human Hoveidaei, Amirhossein Ghaseminejad-Raeini, Sina Esmaeili, Amirmohammad Sharafi, Ali Ghaderi, Kasra Pirahesh, Alireza Azarboo, Basilia Onyinyechukwu Nwankwo, Janet D. Conway

**Affiliations:** 1 Sports Medicine Research Center, Neuroscience Institute, Tehran University of Medical Sciences, Tehran, Iran; 2 School of Medicine, Tehran University of Medical Sciences, Tehran, Iran; 3 Sina University Hospital, Tehran University of Medical Sciences, Tehran, Iran; 4 International Center for Limb Lengthening, Rubin Institute for Advanced Orthopedics, Sinai Hospital of Baltimore, Baltimore, MD USA; 5 Department of Orthopaedic Surgery and Rehabilitation, Howard University Hospital, Washington, DC USA

**Keywords:** Bone graft, Synthetic bone graft, Bone substitute, Autograft alternative, Ankle surgery, Arthrodesis

## Abstract

**Background:**

All orthopaedic procedures, comprising foot and ankle surgeries, seemed to show a positive trend, recently. Bone grafts are commonly employed to fix bone abnormalities resulting from trauma, disease, or other medical conditions. This study specifically focuses on reviewing the safety and efficacy of various bone substitutes used exclusively in foot and ankle surgeries, comparing them to autologous bone grafts.

**Methods:**

The systematic search involved scanning electronic databases including PubMed, Scopus, Cochrane online library, and Web of Science, employing terms like 'Bone substitute,' 'synthetic bone graft,' 'Autograft,' and 'Ankle joint.' Inclusion criteria encompassed RCTs, case-control studies, and prospective/retrospective cohorts exploring different bone substitutes in foot and ankle surgeries. Meta-analysis was performed using R software, integrating odds ratios and 95% confidence intervals (CI). Cochrane's Q test assessed heterogeneity.

**Results:**

This systematic review analyzed 8 articles involving a total of 894 patients. Out of these, 497 patients received synthetic bone grafts, while 397 patients received autologous bone grafts. Arthrodesis surgery was performed in five studies, and three studies used open reduction techniques. Among the synthetic bone grafts, three studies utilized a combination of recombinant human platelet-derived growth factor BB homodimer (rhPDGF-BB) and beta-tricalcium phosphate (β-TCP) collagen, while four studies used hydroxyapatite compounds. One study did not provide details in this regard. The meta-analysis revealed similar findings in the occurrence of complications, as well as in both radiological and clinical evaluations, when contrasting autografts with synthetic bone grafts.

**Conclusion:**

Synthetic bone grafts show promise in achieving comparable outcomes in radiological, clinical, and quality-of-life aspects with fewer complications. However, additional research is necessary to identify the best scenarios for their use and to thoroughly confirm their effectiveness.

**Levels of evidence:**

Level II.

**Supplementary Information:**

The online version contains supplementary material available at 10.1186/s12891-024-07676-8.

## Introduction

Foot and ankle musculoskeletal issues accounts for an enormous part of annual orthopaedic surgical procedures performed all around the world [1–7]. All orthopaedic procedures, comprising foot and ankle surgeries, seemed to show a positive trend, recently [8, 9]. Globally, bone substitutes are needed in about 10% of all orthopedic surgeries. Autogenous bone is the preferred choice because it provides osteoconduction, osteoinduction, and osteogenesis, ensuring compatibility and reducing the risk of disease transmission or rejection. However, to overcome the limitations and scarcity of autogenous bone, recent advancements have introduced several alternative therapeutic approaches such as synthetic bone grafts, local growth factors, and composites [10].

In orthopaedic surgery, bone grafts are frequently used to fix bone abnormalities brought on by trauma, disease, or other medical issues. Due to its capacity to offer all biologic components necessary for a functioning graft, autograft is regarded as the gold standard for bone grafting [11, 12]. Autografts involve moving bone from a particular region of the body—the donor site—to another. Numerous foot and ankle treatments, such as medial ankle instability, osteochondral lesions, arthrodesis, and tibiotalocalcaneal fusion have been investigated with respect to the application of autologous bone transfers [12, 13]. Numerous studies have compared the safety and effectiveness of autologous bone grafts to those of other treatments. According to a comprehensive analysis, autologous bone transplants are safe and have a low incidence of surgical or medical complications [14]. However, there remains some major limitations about utilizing autografts in orthopaedic setting. Donor site challenges such as excessive pain, superficial infection, osteomyelitis, and nerve damage made surgeons think about finding other alternatives [15].

Because of their limitless supply and simplicity in sterilization, synthetic bone grafts have attracted a lot of attention [16, 17]. These alternatives can be divided into three groups: calcium sulfate, tricalcium phosphate, and hydroxyapatite [18]. In contrast to autologous grafts, which call for a second surgical site and might lead to source site complications, synthetic bone grafts allay these worries by requiring no extra surgery. Nonetheless, they have drawbacks like varying rates of resorption and inadequate efficacy in some therapeutic scenarios [17]. Recently, bone graft grafts have been evaluated alongside autografts or allografts in the setting of particular orthopedic operations, such as spinal fusion [19], maxillary sinus augmentation [20], tibial plateau fractures [21], and upper extremity surgery [22]. However, limited evidence exists discussing the application of synthetic bone grafts in foot and ankle surgery (mostly regarding ankle arthrodesis) [15]. A comprehensive systematic review and meta-analysis is definitely demanded to decide which substitute fulfils the criteria to be appropriate for filling bone defects during procedures related to foot or ankle. The present study aimed to systematically review safety and efficacy of different types of bone substitutes in foot and ankle surgeries exclusively compared to autologous bone grafts.

## Materials and methods

The "preferred reporting items for systematic reviews and meta-analyses" (the "PRISMA" statement)" requirements were followed when conducting this meta-analysis [23]. The prospective register of systematic reviews (PROSPERO) records the predefined approach that this review followed (CRD42022372290).

### Search strategy and screening

Electronic databases involving MEDLINE/PubMed, Scopus, Cochrane Central Register of Controlled Trials (CENTRAL), and Web of Science were searched by two independent authors (AG, AMS). A manual search was conducted among publications that were similar to the ones being searched, related articles, and Google Scholar citations. Citation search was updated prior to the final analysis, with the most recent data update occurring on December 20, 2023. The following MeSH headings (Medical Subject Headings) or keyword phrases were employed: Bone substitute, synthetic bone graft, artificial bone, Hydroxyapatite, rhPDGF-BB, Autograft, autologous bone graft, Ankle joint, Foot joints, etc. The search approach is further described in supplementary data, table S1. Using Rayyan, a web-based tool for systematic reviewing, studies were reviewed. Each study was reviewed separately by two reviewers (AG, AMS), who also checked the full-text and removed any duplicates after screening the title and abstract. Studies that met the inclusion-exclusion criteria were chosen. Consensus sessions presided over by the third author (AHH) helped settle any disputes that might have developed between reviewers. Finally, the references of the included articles were checked to ensure that no relevant articles were omitted.

### Inclusion and exclusion criteria

The following inclusion criteria were used to find eligible studies: 1) Participants: Patients who underwent any surgical management needing bone graft due a foot or ankle-related issue; 2) Intervention: Synthetic bone grafts such as Recombinant human platelet-derived growth factor-BB (rhPDGF-BB) and hydroxyapatite-based grafts; 3) Comparison: Autologous bone graft; 4) Outcome: at least one of the following outcomes including postoperative complications, union rate, functional status, and quality of life; 5) Retrospective or prospective cohort studies, case-control studies, or randomized clinical trials (RCTs) were eligible for inclusion. The following exclusion standards were applied: 1) inadequate information to calculate odds ratios or standardized mean differences; 2) Letters, correspondents, pilot studies, reviews and commentaries, technique papers, conference abstracts, animal research, and cadaver studies; 3) Studies without a comparable control group; 4) Studies involving patients undergoing surgical care who required a bone graft in anatomical areas other than the foot and ankle

### Data extraction and Quality assessment

Two researchers (SE, AGR) separately filled out the following data on a pre-created Microsoft Excel sheet before records screening. The information was gathered on demographic characteristics of the patients, such as year of publication, study design, number of participants in both comparison groups, mean age in years, Body mass index (BMI), as well as topic-specific information including bone graft type, mean length of follow-up, complications by detail, pain at the follow-up, functional outcomes including AOFAS score and ATRS score, union rate at the follow-up, and quality of life measures. By the third reviewer (AHH), conflicts were evaluated. Cochrane risk of bias 2 (RoB2) and the ROBINS-I risk assessment tool, respectively, were employed as the criteria for measuring the risk of bias in randomized clinical trials (RCT) and non-RCTs [24]. The following areas were evaluated for bias risk using the RoB2: the randomization process, deviations from intended interventions, missing outcome data, assessment of the outcome, and choice of the reported result. The authors assigned a score of "low," "some concerns," or "high" to each domain. Using the maximum risk associated to any one area as our basis, overall ROB was calculated for each trial [24].

The ROBINS-I tool was modified in accordance with a similarly modified version to better assess risk of bias (RoB) in exposure studies [25], but regardless, the authors adhered to the comprehensive instructions for the ROBINS-I tool [26]. Each study was evaluated in relation to a hypothetical target randomized trial, with differences from the target trial being viewed as bias. By responding to signaling questions and critically mirroring each domain against a set of predetermined criteria, seven bias domains, including confounding, selection of participants, classification of diet groups, departures from baseline diet groups, missing data, measurement of outcomes, and selection of the reported results were evaluated.

Each domain could receive a "low," "moderate," "serious," "critical," or "no information" RoB rating, or a "no information" RoB rating. After that, each study was given an overall RoB assessment (study-level assessment) based on a different set of criteria. According to ROBINS, the overall RoB for a study was determined by assigning the most severe RoB judgment to each domain.

### Data analyses

The data analyses and the ensuing data synthesis were conducted using R software, version 4.2.2 (R Foundation for Statistical Computing, Vienna, Austria; http://www.R-project.org). By inverse-variance method, standardized mean differences (SMD) was computed to evaluate continuous outcomes [27]. The Mantel-Haenszel method was used to produce the odds ratio (OR) and associated 95% confidence intervals (CI) as the effect estimate for all categorical data. Depending on the level of heterogeneity, a fixed-effect model and a random-effects model was employed to pool study-specific effect estimates for high heterogeneity and low heterogeneity. The Q-test and I2 were used to assess statistical heterogeneity. Low, moderate, and high heterogeneity were deemed to be represented by I2 values of 25, 50, and 75%, respectively [28]. If *P*>0.1 and I2<50%, a fixed effect model was used; otherwise, a random-effect model was applied [29]. To assess the publication bias, Egger's test was employed [30]. For all data analyses, with the exception of heterogeneity, a value of *P*<0.05 was taken as showing statistical significance, and all tests were two-sided.

## Results

### Study selection

**Fig. 1:**
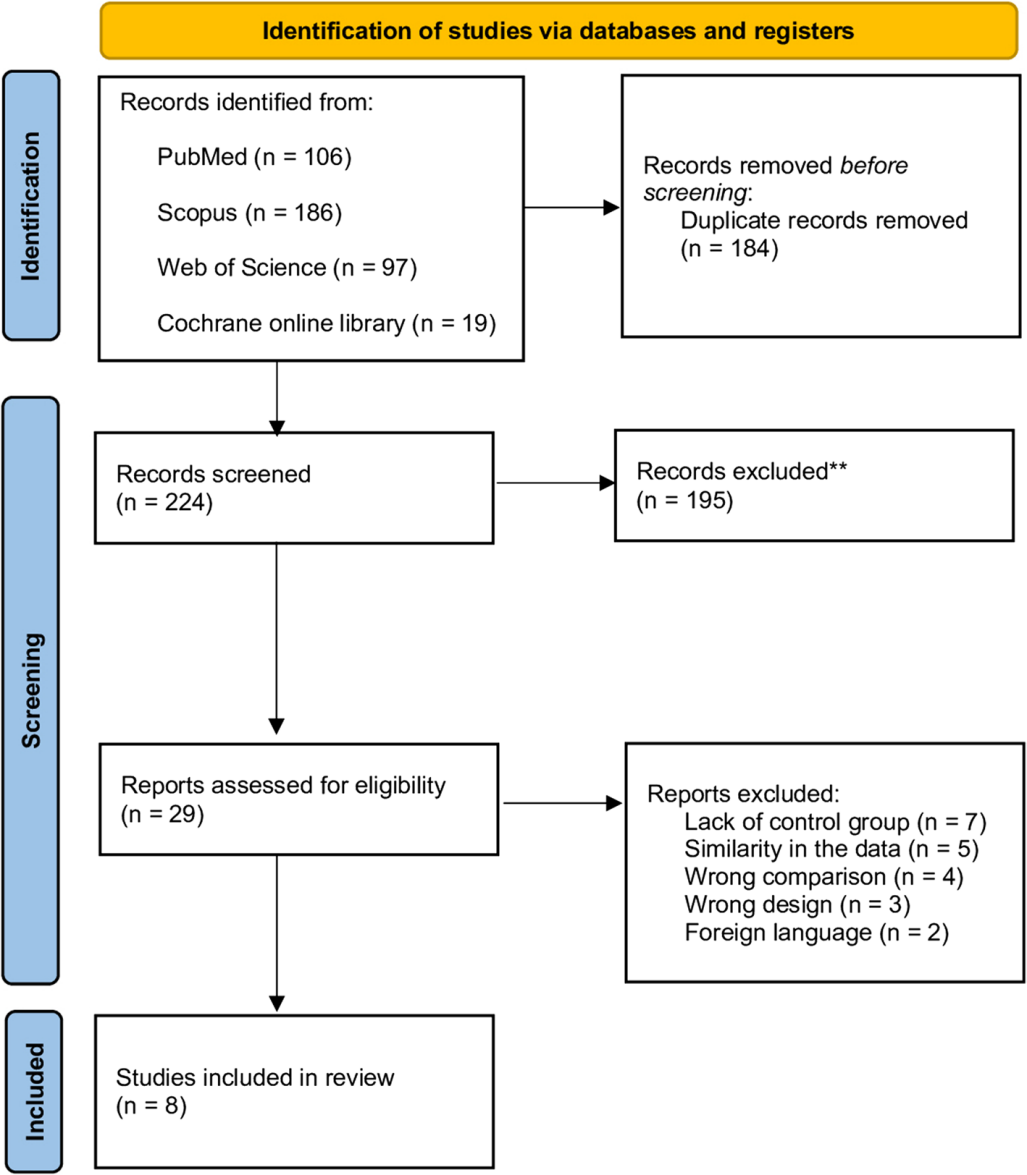
PRISMA flow diagram

After collecting the search results from various databases and eliminating duplicate entries, a total of 224 articles were subjected to initial screening based on their titles and abstracts. Subsequently, the full text of 29 articles was assessed. Out of these, 21 articles were excluded due to issues such as the wrong design or comparison group, foreign language, absence of a control group, as well as similarity in the data. Eventually, eight articles were deemed unique and non-repetitive, and they were consequently incorporated into this systematic review (Fig. 1) [31–38]. Digiovanni et al. conducted a study in 2016 [39]. However, due to the similarity of the study population with their 2013 publication, and the 2013 study being more suitable for our current research, The authors have chosen to include the 2013 study in our systematic review.

### Risk of *bias*

Among the eight included articles, five of them are randomized clinical trials (RCTs), while the remaining studies are either retrospective or prospective cohorts. For the evaluation of the risk of bias, the ROBINs-I tool was used for cohorts, and the RoB 2 tool was employed for RCTs. The findings of the risk of bias assessment are summarized in supplementary data, Tables S2 and S3.

### Bassline characteristics

A total of eight articles included in this review analyzed the data of 894 patients, 497 (55.6%) of them received synthetic bone grafts and 397 (44.4%) patients underwent surgical procedures using autologous bone grafts. Two studies were conducted collaboratively by researchers in the USA and Canada [31, 33]. One study was carried out in each of the USA [32], Canada [35], and Italy [34], while three studies were conducted in China [36–38]. The mean age ranged from 41.7 to 57.5 years, with a total of 484 males and 396 females included in the studies. Four articles reported body mass index (BMI), with a mean range of 20.26 to 31.4. Follow-up duration ranged from 9 to 145 months (Table 1).

**Table 1:** Baseline characteristics of included studies

**Author, year**	**Country**	**Study design**	**Total population, n**	**Age, years**	**Male/Female**	**BMI, kg/m**^**2**^	**Smoking**	**Follow-up duration, months**	**Lost to follow-up, n**
Digiovanni et al., 2013 [33]	USA & Canada	Prospective, multicenter, non-inferiority, pivotal RCT	Synthetic: 260 (394 joints) Autologous: 137 (203 joints)	Synthetic: 56.2 (19.8-86.2) Autologous: 57.5 (20.3-82.2)	Synthetic: 123/137 Autologous: 78/59	NM	NM	13	NM
Daniels et al., 2019 [31]	USA & Canada	Blinded, noninferiority RCT	Synthetic: 132 (132 joints) Autologous: 167 (167 joints)	Synthetic: 53.5 ± 14.7 Autologous: 56.2 ± 14.0	Synthetic: 70/62 Autologous: 86/81	Synthetic: 31.2 ± 6.1 Autologous: 31.4 ± 5.7	Synthetic: 71 Autologous: 84	13	3
Digiovanni et al., 2011 [32]	USA	Prospective, Multicenter, Feasibility RCT	Synthetic: 14 (14 joints) Autologous: 6 (6 joints)	Synthetic: 55.2 Autologous: 43.7	Synthetic: 4/10 Autologous: 5/1	Synthetic: 29.8 Autologous: 30.5	Synthetic: 7 Autologous: 1	9	NM
Fortina et al., 1998 [34]	Italy	Cohort	Synthetic: 9 (11 joints) Autologous: 5 (5 joints)	NM	NM	NM	NM	Synthetic: 34 ± 12.9 Autologous: 145 ± 43.7	NM
Glazebrook et al., 2013 [35]	Canada	Prospective, pilot RCT	Synthetic: 12 (12 joints) Autologous: 12 (12 joints)	Synthetic: 54.5 ± 14.3 Autologous: 57.9 ± 14.0	Synthetic: 5/7 Autologous: 10/2	Synthetic: 30.8 ± 5.5 Autologous: 30.3 ± 4.7	Synthetic: 1 Autologous: 2	12	NM
Lian et al., 2013 [36]	China	Retrospective cohort	Synthetic: 24 (24 joints) Autologous: 24 (24 joints)	Total: 46	Synthetic: 16/8 Autologous: 16/8	NM	Synthetic: 0 Autologous: 0	17 (12-24)	NM
Pan et al., 2018 [37]	China	Prospective, single blind RCT	Synthetic: 30 (30 joints) Autologous: 30 (30 joints)	Synthetic: 41.8 ± 10.0 Autologous: 41.7 ± 7.7	Synthetic: 27/3 Autologous: 28/2	NM	NM	17.2 ± 3.0	NM
Wan et al., 2020 [38]	China	Retrospective cohort	Synthetic: 16 (16 joints) Autologous: 16 (16 joints)	Synthetic: 48.44 ± 6.42 Autologous: 47.94 ± 6.27	Synthetic: 8/8 Autologous: 8/8	Synthetic: 20.63 ± 1.18 Autologous: 20.26 ± 1.09	Synthetic: 0 Autologous: 0	22 (18-28)	1

*RCT* Randomized Controlled Trial, *n* Number, *NM* Not Mentioned

In a total of eight studies, two different surgical techniques were utilized for various orthopedic purposes. Among these, arthrodesis surgery was employed in five studies [31–33, 35, 38], while three studies used open reduction [34, 36, 37]. In three of these studies, synthetic bone grafts consisting of a combination of recombinant human platelet-derived growth factor BB homodimer (rhPDGF-BB) and beta-tricalcium phosphate (β-TCP) collagen were employed [31–33]. The remaining four studies, except for Wan et al.'s study which did not specify details in this regard, employed hydroxyapatite compounds. All of these studies utilized entirely artificial grafts as synthetic bone substitutes. These surgical interventions targeted a range of bones and joints, including the ankle, calcaneus, subtalar, calcaneocuboid, and talonavicular (Table 2).

**Table 2:** Diagnostic and surgical technique details

**Author, year**	**Surgical procedure**	**Bone substitute type**	**Affected bone and joint**	**Primary diagnosis**	**Operation time, min**	**Blood loss, ml**
Digiovanni et al., 2013 [33]	Arthrodesis	rhPDGF-BB/β-TCP	NM	primary osteoarthritis: 34.3% posttraumatic arthritis or deformity: 48.2% rheumatoid arthritis: 6.7%	NM	NM
Daniels et al., 2019 [31]	Arthrodesis	rhPDGF-BB/β-TCP	Ankle: Synthetic: 31 (23.5%) Autologous: 46 (27.5%) Subtalar: Synthetic: 52 (39.4%) Autologous: 59 (35.3%) Calcaneocuboid: Synthetic: 3 (2.3%) Autologous: 0 Talonavicular: Synthetic: 6 (4.6%) Autologous: 9 (5.4%) Double fusion: Synthetic: 21 (15.9%) Autologous: 17 (10.2%) Triple Arthrodesis: Synthetic: 19 (14.4%) Autologous: 36 (21.6%)	Posttraumatic arthritis or deformity: Synthetic: 58 (43.9%) Autologous: 68 (40.7%) Primary osteoarthritis: Synthetic: 60 (45.5%) Autologous: 65 (38.9%) Rheumatoid arthritis: Synthetic: 8 (6.1%) Autologous: 12 (7.2%) Ankylosing spondylitis: Synthetic: 0 Autologous: 1 (0.6%) Congenital or acquired deformity: Synthetic: 4 (3.0%) Autologous: 6 (3.6%) Other: Synthetic: 2 (1.5%) Autologous: 15 (9.0%)	NM	NM
Digiovanni et al., 2011 [32]	Arthrodesis	rhPDGF-BB/β-TCP	Ankle: Synthetic: 5 (36%) Autologous: 2 (33%) Subtalar: Synthetic: 3 (21%) Autologous: 1 (17%) Triple Arthrodesis: Synthetic: 6 (43%) Autologous: 3 (50%)	Primary Arthritis: Synthetic: 2 (14%) Autologous: 1 (17%) Rheumatoid Arthritis: Synthetic: 2 (14%) Autologous: 0 Post-traumatic Arthritis: Synthetic: 9 (65%) Autologous: 5 (83%)	Synthetic: 118 Autologous: 144	NM
Fortina et al., 1998 [34]	ORIF	Hydroxyapatite	Calcaneus	Displaced intra-articular fractures	NM	NM
Glazebrook et al., 2013 [35]	Arthrodesis	B2A peptide-coated ceramic granules	Ankle: Synthetic: 6 (50%) Autologous: 8 (66.7%) Subtalar: Synthetic: 5 (41.7%) Autologous: 4 (33.3%) Talonavicular: Synthetic: 1 (8.3%) Autologous: 0 (0%)	NM	Synthetic: 95 ± 24 Autologous: 105 ± 31	Synthetic: 35 ± 58 Autologous: 58 ± 78
Lian et al., 2013 [36]	ORIF	Mineralized collagen: self-assembly of collagen triple helices and hydroxyapatite mixed with polylactic acid	Calcaneus	Closed displaced intra-articular fractures	Synthetic: 80 (50-100) Autologous: 106 (70-120)	NM
Pan et al., 2018 [37]	ORIF	Mineralized collagen: composed of arranged collagen and nano-sized hydroxyapatite	Calcaneus	Closed calcaneal fracture	Synthetic: 75.90 ± 4.75 Autologous: 86.93 ± 5.26	Synthetic: 231 ± 444 Autologous: 266 ± 55
Wan et al., 2020 [38]	Arthrodesis	Artificial bone graft	Subtalar	Traumatic arthritis of the subtalar joint	NM	NM

*rhPDGF-BB* Recombinant human platelet-derived growth factor-BB, *β-TCP* β-tricalcium phosphate, *ORIF* Open Reduction and Internal Fixation, *NM* Not Mentioned

### Arthrodesis

#### rhPDGF-BB/β-TCP bone graft

**Fig. 2:**
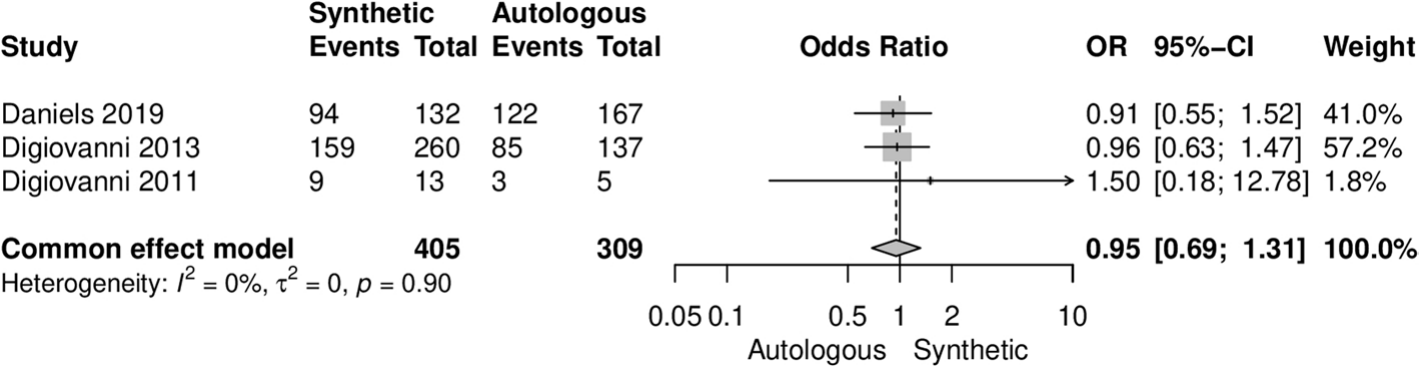
Forest plot illustrating the fusion rate assessed by CT scans in the synthetic (rhPDGF-BB/β-TCP bone graft) and autologous groups

In three out of five studies, the rhPDGF-BB/β-TCP material was utilized as a synthetic bone graft [31–33]. In the studies, before patients received this synthetic graft material, the ingredients (rhPDGF-BB 0.3 mg/mL solution and β-TCP-collagen matrix) were combined and allowed at least 10 minutes to fully saturate before insertion at the fusion site using a cannula. A meta-analysis of these three studies revealed no significant difference between the synthetic and autologous groups in terms of CT fusion rate (OR [95%CI] = 0.95 [0.69-1.31], I^2^ = 0%) (Fig. 2). Regarding radiographic union rate, an analysis of all joints' three aspects union in Digiovanni et al.'s study indicated a significant difference between the two groups, favoring the synthetic bone graft (48.5% vs. 44.3%, *P* < 0.001) (Table 3) [33].

**Table 3:** Radiological outcomes

**Author, year**	**Fusion success rate**	**CT-scan union**	**Radiographic union**	**Time to union, weeks**	**Bohler's angle**	**Gissane’s angle**	**Calcaneus height**
Digiovanni et al., 2013 [33]	Full-Complement CT fusion rates (24 weeks): Synthetic: 159/260 (61.2%) Autologous: 85/137 (62.0%) All-Joints CT fusion rates (24 weeks): Synthetic: 262/394 (66.5%) Autologous:127/203 (62.6%)	NM	Full-Complement three aspects union rate: Synthetic: 96/260 (36.9%) Autologous: 50/137 (36.5%) Full-Complement two aspects union rate: Synthetic: 184/260 (70.8%) Autologous: 103/137 (75.2%) All-Joints three aspects union rate: Synthetic: 191/394 (48.5%) Autologous: 90/203 (44.3%) All-Joints two aspects union rate: Synthetic: 304/394 (77.2%) Autologous: 158/203 (77.8%)	NM	NM	NM	NM
Daniels et al., 2019 [31]	CT full complement fusion (36+ weeks): Synthetic: 94/132 (71.0%) Autologous: 122/167 (73.1%)	NM	Full-Complement three aspects union rate: Synthetic: 46/132 (35.0%) Autologous: 56/167 (33.7%) Full-Complement two aspects union rate: Synthetic: 100/132 (75.9%) Autologous: 132/167 (78.9%)	NM	NM	NM	NM
Digiovanni et al., 2011 [32]	X-ray Fusion (12 weeks): Synthetic: 5/12 (42%) Autologous: 1/3 (33%) CT Fusion (12 weeks): Synthetic: 9/13 (69%) Autologous: 3/5 (60%) X-ray Fusion (36 weeks): Synthetic: 10/13 (77%) Autologous: 3/6 (50%)	NM	NM	NM	NM	NM	NM
Fortina et al., 1998 [34]	NM	NM	NM	NM	Contralateral normal foot: 30.9° ± 5.8° Synthetic: 20.5° ± 8.2° Autologous: 23.4° ± 4.9°	Contralateral normal foot: 121.3° ± 5.3° Synthetic: 125.3° ± 10° Autologous: 122° ± 4.5°	Contralateral normal foot: 49.5 ± 3.1 Synthetic: 45 ± 4.9 Autologous: 46 ± 5.6
Glazebrook et al., 2013 [35]	9 months: Synthetic: 12/12 (100.0%) Autologous: 8/12 (66.7%)	9 months: Synthetic: 10/12 (83.3%) Autologous: 7/12 (58.3%)	NM	NM	NM	NM	NM
Lian et al., 2013 [36]	NM	NM	NM	Synthetic: 8.3 Autologous: 7.9	NM	NM	NM
Pan et al., 2018 [37]	NM	NM	NM	Synthetic: 10.03 ± 1.73 Autologous: 9.80 ± 1.75	Synthetic: pre-op: 4.70° ± 6.52° post-op: 30.27° ± 3.35° Autologous: pre-op: 3.77° ± 7.15° post-op: 29.70° ± 3.11°	Synthetic: pre-op: 137.17° ± 8.83° post-op: 116.17° ± 5.36° Autologous: pre-op: 137.70° ± 7.62° post-op: 117.40° ± 4.70°	Synthetic: pre-op: 39.13 ± 3.26 post-op: 47.77 ± 2.93 Autologous: pre-op: 38.20 ± 2.94 post-op: 46.47 ± 2.52

*NM* Not Mentioned

**Fig. 3:**
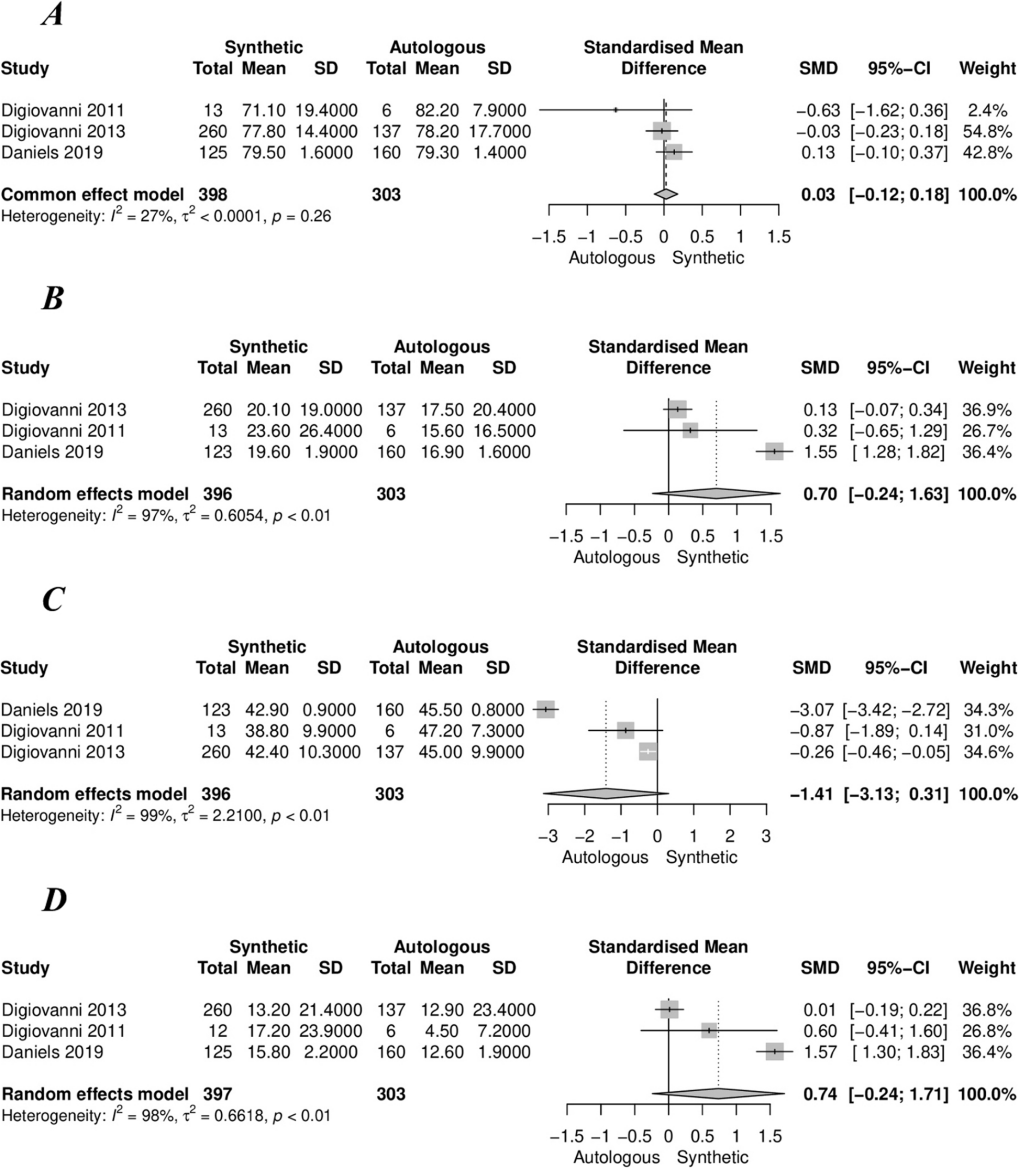
Forest plots illustrating **A**) American Orthopaedic Foot & Ankle Society (AOFAS) functional score, **B**) Foot Function Index (FFI), **C**) Short-Form 12 (SF-12) Physical Component Summary (PCS), and **D**) Visual Analog Scale (VAS) in the synthetic (rhPDGF-BB/β-TCP bone graft) and autologous groups

Furthermore, no significant differences were observed in terms of American Orthopaedic Foot & Ankle Society (AOFAS) functional score (SMD [95%CI] = 0.03 [-0.13-0.18], I^2^ = 27%) (Fig. 3A), Foot Function Index (FFI) (SMD [95%CI] = 0.70 [-0.24-1.63], I^2^ = 97%) (Fig. 3B), and Short-Form 12 (SF-12) Physical Component Summary (PCS) (SMD [95%CI] = -1.41 [-3.13-0.31], I^2^ = 99%) (Fig. 3C). The visual analog scale (VAS) (pain) at the fusion site did not exhibit a significant difference (OR [95%CI] = 0.74 [-0.24-1.71], I^2^ = 98%) (Fig. 3D).

**Fig. 4:**
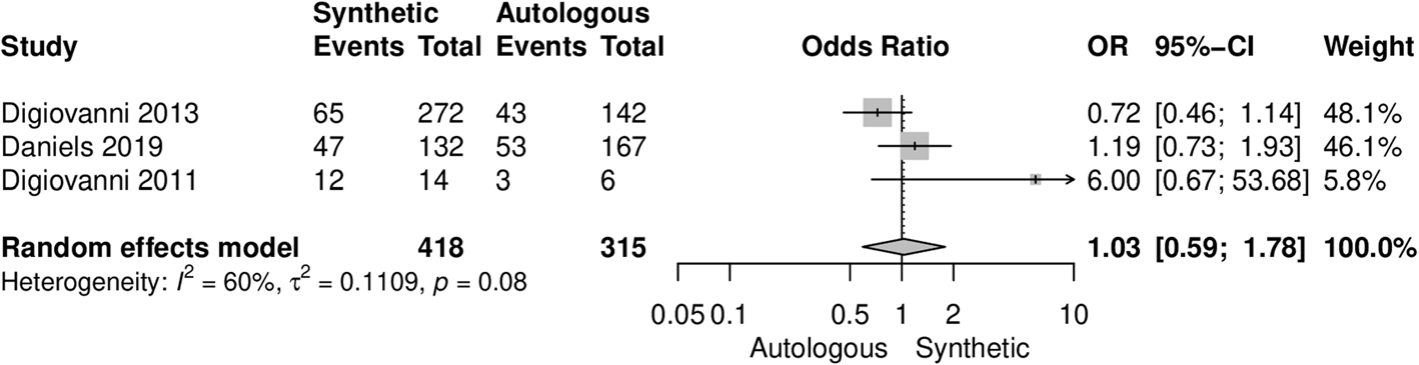
Forest plot illustrating the surgical complications rate in the synthetic (rhPDGF-BB/β-TCP bone graft) and autologous groups

There were no significant differences in surgical complications between the two groups (OR [95%CI] = 1.03 [0.59-1.78], I^2^ = 60%) (Fig. 4). Additionally, Digiovanni et al. found no significant disparities in the occurrence of serious adverse events (*P* = 0.201), device-related adverse events (*P* = 0.354), or serious complications (*P* = 0.654) between the two groups [33]. similarly, Daniels et al. also reported no notable distinctions in the occurrence of serious adverse events (*P* = 1.00), device-related adverse events (*P* = 0.741), serious complications (*P* = 0.808), or infections (*P* = 0.127) between autologous or synthetic bone grafts (Table 5) [31].

#### Other bone substitutes

Glazebrook et al. conducted a study on patients who underwent arthrodesis procedures, with 12 cases using B2A peptide-coated ceramic granules as a bone graft and 12 cases utilizing autologous grafts. They employed B2A peptide-coated ceramic granules in kit form, each containing a vial of lyophilized B2A peptide and porous granules composed of 80% tricalcium phosphate and 20% hydroxyapatite. They reported a 100% fusion success rate in the synthetic group compared to 66.7% in the autologous group, with an 83% complete union rate in the synthetic group and 58.3% in the autologous group (Table 3). At 6 months, 7 out of 12 subjects in both groups experienced a decrease in pain scores of at least 30%, while 1 out of 12 subjects in both groups had an increased pain score (Table 4). Graft site pain was reported by 2 patients at 6 months, and 2 patients in the synthetic group showed wound infections (Table 5) [35].

**Table 4:** Pre- and Postoperative scores

**Author, year**	**Preoperative clinical scores (VAS, AOFAS, FFI, SF-12, …)**	**Post-operative pain**	**Post-operative AOFAS score**	**Post-operative FFI score**	**Other post-operative functional scores**	**Post-operative SF-12 score**
Digiovanni et al., 2013 [33]	NM	VAS pain weight-bearing: Synthetic: 15.6 ± 22.4 Autologous: 15.8 ± 25.2 VAS pain at fusion site: Synthetic: 13.2 ± 21.4 Autologous: 12.9 ± 23.4 graft harvest site pain: Synthetic: NM Autologous: 12n	Synthetic: 77.8 ± 14.4 Autologous: 78.2 ± 17.7	Synthetic: 20.1 ± 19.0 Autologous: 17.5 ± 20.4	NM	PCS: Synthetic: 42.4 ± 10.3 Autologous: 45.0 ± 9.9
Daniels et al., 2019 [31]	VAS pain weightbearing: Synthetic: 72.0 ± 22.3 Autologous: 69.9 ± 22.0 VAS pain at fusion site: Synthetic: 49.8 ± 26.3 Autologous: 51.7 ± 26.4 AOFAS: Synthetic: 43.3 ± 17.2 Autologous: 43.6 ± 16.8 FFI: Synthetic: 50.6 ± 18.4 Autologous: 50.0 ± 15.1 SF-12 (PCS): Synthetic: 30.8 ± 8.4 Autologous: 31.2 ± 8.3	VAS pain weight-bearing: Synthetic: 16.6 ± 2.4 (*n*=124) Autologous: 15.9 ± 2.1 (*n*=157) VAS pain at fusion site: Synthetic: 15.8 ± 2.2 (*n*=125) Autologous: 12.6 ± 1.9 (*n*=160)	Synthetic: 79.5 ± 1.6 (*n*=125) Autologous: 79.3 ± 1.4 (*n*=160)	Synthetic: 19.6 ± 1.9 (*n*=123) Autologous: 16.9 ± 1.6 (*n*=160)	NM	PCS: Synthetic: 42.9 ± 0.9 (*n*=123) Autologous: 45.5 ± 0.8 (*n*=160)
Digiovanni et al., 2011 [32]	VAS pain at fusion site: Synthetic: 32.0 ± 32.66 (*n*=13) Autologous: 36.3 ± 39.20 (*n*=6) AOFAS: Synthetic: 39.2 ± 17.93 Autologous: 36.3 ± 24.35 FFI: Synthetic: 47.4 ± 15.59 Autologous: 45.9 ± 15.59 SF-12 (PCS): Synthetic: 34.1 ± 7.45 Autologous: 38.2 ± 11.47 (*n*=6) SF-12 (MCS): Synthetic: 49.8 ± 11.16 (*n*=13) Autologous: 47.5 ± 10.65 (*n*=6)	VAS pain at fusion site: Synthetic: 17.2 ± 23.91 (n=12) Autologous: 4.5 ± 7.15 (*n*=6) graft harvest site (Post surgery): Synthetic: NM Autologous: 45.3 ± 39.37 (*n*=5) graft harvest site (12 weeks): 14.9 ± 26.52 (*n*=5)	Synthetic: 71.1 ± 19.38 (*n*=13) Autologous: 82.2 ± 7.86 (*n*=6)	Synthetic: 23.6 ± 26.35 (*n*=13) Autologous: 15.6 ± 16.45 (*n*=6)	NM	PCS: Synthetic: 38.8 ± 9.86 (*n*=13) Autologous: 47.2 ± 7.27 (*n*=6) MCS: Synthetic: 49.8 ± 11.16 (*n*=13) Autologous: 47.5 ± 10.65 (*n*=6)
Fortina et al., 1998 [34]	NM	Creighton Nebraska Health Foundation scoring system: Synthetic: 27.3 ± 2.6 Autologous: 29 ± 2.4	NM	NM	Creighton Nebraska Health Foundation scoring system: Synthetic: 90-100: 5n, 80-89: 3n, 65-79: 3n, <64: 0n Autologous: 90-100: 4n, 80-89: 0n, 65-79: 1n, <64: 0n	NM
Glazebrook et al., 2013 [35]	NM	Decreased at least 30% (6 months): Synthetic: 7/12 Autologous: 7/12 Increased: Synthetic: 1/12 Autologous: 1/12 graft harvest site pain (6 months): Synthetic: NM Autologous: 2/12	NM	NM	NM	NM
Lian et al., 2013 [36]	NM	graft harvest site pain (12 months): Synthetic: NM Autologous: 3/24	NM	NM	Maryland foot score (12 months): Synthetic: 90 ± 12 Autologous: 86 ± 10	NM
Pan et al., 2018 [37]	NM	NM	Synthetic: 88.37 ± 3.61 Autologous: 88.37 ± 4.74	NM	NM	NM
Wan et al., 2020 [38]	VAS: Synthetic: 6.19 ± 0.83 Autologous: 6.13 ± 0.89 AOFAS: Synthetic: 51.44 ± 8.62 Autologous: 51.25 ± 8.93	VAS: Synthetic: 1.56 ± 0.63 Autologous: 1.50 ± 0.73	Synthetic: 74.18 ± 10.54 Autologous: 74.06 ± 9.31	NM	NM	NM

*VAS* Visual Analog Scale, *AOFAS* American Orthopaedic Foot and Ankle Society, *FFI* Foot Function Index, *SF* Short Form, *PCS* Physical Component Summary, *MCS* Mental Component Summary, *NM* Not Mentioned

**Table 5:** Postoperative complications after bone graft augmentation

**Author, year**	**Serious treatment emergent adverse events, n (%)**	**Device-related treatment emergent adverse events, n (%)**	**Surgical complications, n (%)**	**Serious complications, n (%)**	**Infection, n (%)**	**Other complications, n (%)**
Digiovanni et al., 2013 [33]	Synthetic: 28/272 (10.3%) Autologous: 21/142 (14.8%)	Synthetic: 6/272 (2.2%) Autologous: 6/142 (4.2%)	Synthetic: 65/272 (23.9%) Autologous: 43/142 (30.3%)	Synthetic: 14/272 (5.1%) Autologous: 9/142 (6.3%)	NM	NM
Daniels et al., 2019 [31]	Synthetic: 17/132 (12.7%) Autologous: 25/167 (15.0%)	Synthetic: 3/132 (2.3%) Autologous: 6/167 (3.6%)	Synthetic: 47/132 (35.6%) Autologous: 53/167 (31.1%)	Synthetic: 8/132 (6.1%) Autologous: 10/167 (6.0%)	Synthetic: 27/132 (20.5%) Autologous: 26/167 (15.6%)	NM
Digiovanni et al., 2011 [32]	Synthetic: 0 Autologous: 0	Synthetic: 0 Autologous: 0	Synthetic: 12/14 (85.7%) Autologous: 3/6 (50.0%)	Synthetic: 0 Autologous: 0	NM	NM
Fortina et al., 1998 [34]	NM	NM	NM	NM	Synthetic: 0 Autologous: 0	No reflex sympathetic dystrophies or thrombophlebitis
Glazebrook et al., 2013 [35]	NM	NM	NM	NM	Synthetic: 2 Autologous: 0	Synthetic: 5n (Hemorrhoids: 1,Transient liver enzyme increase: 2, Wound breakdown: 1, Lateral ankle pain: 1) Autologous: 4n (Transient liver enzyme increase: 2, Above knee DVT operation: 1, Detached retina: 1)
Lian et al., 2013 [36]	NM	NM	NM	NM	Total: 3	NM
Pan et al., 2018 [37]	NM	NM	NM	NM	NM	Wound complications: Synthetic: 2 Autologous: 2 Rejection: Synthetic: 0 Autologous: 0 Donor site complications: Synthetic: 0 Autologous: 8
Wan et al., 2020 [38]	NM	NM	NM	NM	NM	Incision complication: Synthetic: 0 Autologous: 0

*NM* Not Mentioned, *n* Number

In a study led by Wan et al., they examined the outcomes of arthroscopy-assisted arthrodesis in two distinct groups, each consisting of 16 patients (not specified bone substitute type). Postoperative VAS and AOFAS scores did not show significant differences between the synthetic and autologous groups (*P* = 0.990 and 0.995, respectively) (Table 4) [38].

#### Open reduction and internal fixation (ORIF)

ORIF was the main surgical procedure in three papers [34, 36, 37]. In the study conducted by Lian et al., they utilized mineralized collagen as a synthetic bone graft, which was prepared in two main steps. Firstly, mineralized type I collagen fibrils were formed through the self-assembly of collagen triple helices and hydroxyapatite (HA). HA crystals nucleated and developed within collagen helices, a process regulated by collagen fibers. Secondly, mineralized type I collagen fibrils were combined with a polylactic acid solution to create mineralized collagen through comprehensive freeze-drying. The resulting product was then cut into small granules and sterilized. They discovered that there was no statistically significant difference in the time to union between the synthetic and autologous groups (8.3 and 7.9 weeks, respectively) (*P* > 0.05) [36]. Similarly, in Pan et al.'s study, no significant difference was observed, with values of 10.03 ± 1.73 and 9.80 ± 1.75 for the two groups, respectively (*P* = 0.606) (Table 3) [37]. The artificial mineralized collagen employed by them constituted a biomimetic bone graft, comprising arranged collagen and nano-sized hydroxyapatite.

Moreover, two of these studies also investigated radiographic angles, specifically Bohler and Gissane angles [34, 37]. The Bohler angle, discerned from a lateral foot radiograph, is determined by the angle formed between a line connecting the highest points of the anterior process of the calcaneus and the posterior articular facet, and another line linking the highest point of the posterior articular facet with the apex of the calcaneal tuberosity. Gissane angle is determined by tracing lines along the superior surfaces of the anterior process and the posterior facet of the calcaneus, culminating at the calcaneal sulcus. The Bohler angle is expected to range from 25° to 40°, while the Gissane angle typically falls between 125° and 145°, although there may be some variation about the standard normal range [40].

Both the Bohler and Gissane angles play pivotal roles in evaluating the severity of calcaneal fractures, with surgical intervention aiming to restore these angles to their standard values. Remarkably, both referenced studies found that post-surgery, these angles fell within the accepted normal range. Moreover, in Pan et al.'s investigation, no statistically significant distinction was observed between the two study groups in terms of postoperative angles (*P* < 0.05) (Table 3).

In Fortina et al.'s study, clinical examinations were carried out using the Creighton-Nebraska Health Foundation scoring system. The results showed no significant difference in residual pain levels between the synthetic and autologous groups, with values of 27.3 ± 2.6 and 29 ± 2.4, respectively (*P* > 0.05) [34]. Similarly, Lian et al. utilized the Maryland foot score to assess clinical outcomes, revealing a consistent absence of any notable difference between the two groups. Their findings indicated scores of 12 ± 90 and 10 ± 86 for the respective groups, with no statistically significant distinction evident (*P* > 0.05) [36]. Pan et al.'s study revealed similar findings, indicating that there was no statistically significant distinction in AOFAS scores (*P* = 0.071) between the synthetic group (88.37 ± 3.61) and the autologous group (88.37 ± 4.74) (Table 4) [37].

In Fortina et al.'s study, no instances of septic complications, reflex sympathetic dystrophies, or thrombophlebitis were reported [34]. In contrast, Lian et al. found that three out of 48 patients from both groups experienced wound infections in their study [36]. Conversely, Pan et al.'s investigation showed a comparable incidence of wound complications and rejections between the two groups, with eight out of 30 patients in the autologous group experiencing complications at the donor site [37] (Table 5).

## Discussion

In the current systematic review, the authors found comparable complications, outcomes in radiological, and clinical measures between autograft and synthetic bone grafts. Autogenous bone graft has several advantages, including histocompatibility, osteogenecity, osteoconductive and osteoinductive properties, and no risk of disease transmission [41]. However, acquiring the autograft from the patient is an additional operation posing several complications related to the bone harvesting from donor site. Complications including blood loss, chronic pain at donor site, infection, nerve injury, and amplified surgical duration and expenses may occur due to autograft bone harvesting [41–44]. Furthermore, the patient's age, body mass index (BMI), gender, and overall health status can influence both the quality and quantity of accessible autograft materials [45]. On the other hand, synthetic bone grafts eliminate the potential risks associated with autografts. While synthetic bone grafts may not possess all the benefits of autografts, they have demonstrated favorable results in numerous instances, with potential for further enhancement.

Although the use of bone grafts in ankle arthrodesis is controversial, it is still widely used [46]. The authors found no differences when comparing the autograft with synthetic bone graft in terms of fusion, radiographic union, functional outcome, pain, and complications. As the most frequently used synthetic bone graft among included studies, rhPDGF-BB/β-TCP achieved comparable outcomes to autograft in arthrodesis. Since promoting osseous fusion is the primary goal of using grafts in arthrodesis [13], synthetic grafts can be considered as suitable alternatives based on our results.

Synthetic grafts typically consist of two components: a scaffold and a growth factor. β-TCP's calcium-to-phosphate ratio is similar to that of natural bone mineral, making it suitable for use as a scaffold. It is biocompatible and biodegradable, providing an osteoconductive matrix at the fusion site. Among various homodimers, PDGF-BB has been shown to be particularly important for bone regeneration, promoting mitogenesis, chemotaxis, extracellular matrix formation, and vascularization. Research has demonstrated that rhPDGF-BB can enhance the proliferation of various cell types and osteogenesis, further encouraging bone formation in fractures or defects. Recently, the combination of rhPDGF-BB and β-TCP has been used for ankle and foot fusion due to its exceptional bone healing properties [47–51]. Although, other types of synthetic grafts also achieved similar outcomes, further studies with larger sample sizes may reveal potential differences attributed to the additional osteoinductive properties. Nevertheless, achieving satisfactory results depends on using materials with properties closely resembling those of the autograft, including osteoconductivity, osteoinductivity, biocompatibility, and appropriate biomechanical properties. These grafts should mimic the structure of natural bone, support new bone growth, integrate well with existing tissues, and degrade at a rate that matches new bone formation [52, 53].

In foot and ankle procedures with ORIF, synthetic bone grafts also demonstrated similar outcomes to autografts. Bone grafts in ORIF of foot and ankle are usually used with aim of providing a mechanical support and to maintain alignment [54]. Two studies reporting Gissane and Bohler angles demonstrated restoration to standard values in both graft types. Moreover, time to union, as an important parameter in ORIF, was similar in both groups in 2 studies. Overall, no significant difference was found in radiographical, clinical, and pain-related outcomes and complications between the two groups. In fact, similarity of outcomes in terms of complications was regardless of the donor site morbidities and pain, which favors the use of synthetic bone grafts. Importantly, use of synthetic grafts aids surgeons in filling irregular defects in trauma surgeries, without the limitation posed by available autograft to be safely harvested [37].

A longer incorporation time for grafts which will be placed in anatomically weight-bearing sites demands higher structural stability, which in turn may limit the scaffold’s osteoconductive properties, further lengthening the incorporation time [55, 56]. Hence, the best option should be chosen considering this tradeoff.

Rapid incorporation of the graft to the host site, which is usually the case with foot and ankle surgeries, comes with a lack of durable structure to maintain osteoconductive and osteogenic properties of the graft. Cancellous bone grafts are therefore used most commonly in these surgeries [12]. Based on our review, procedures requiring grafts for the aforementioned purpose responded well to the use of synthetic bone grafts, highlighting the osteoconductive properties of these materials with minimal side effects. However, when structural stability (especially during the initial stages) becomes one of the main objectives, synthetic grafts are not functionally sufficient. Although outcomes such as pain and complications can still be similar, functional requirements of the procedure must be met in order to prevent revisions and validate the utilization of synthetic grafts. Newer synthetic grafts developed to overcome this drawback are necessary to replace autografts in procedures requiring structurally durable grafts.

Beyond assessing the risks and benefits, it is crucial for surgeons, hospitals, insurers, and patients to evaluate the economic impact of new technologies. Research has shown that using autografts incurs significant resource use, including extra operating room time, higher costs for supplies and personnel, additional medications, extended hospital stays, donor site complications, and both immediate and long-term side effects post-harvest [10, 57]. Moving forward, a critical comparison of the costs associated with autografts and synthetic bone grafts is necessary.

Our review was subject to certain limitations. While the majority of findings were in the same direction, the included studies were highly heterogenous, necessitating the use of random effects models in some of our analyses. Also, given the broad array of synthetic bone graft alternatives, the limited volume of research studies comparing their efficacy, either against autograft or within their category, necessitates cautious interpretation of our review's findings. Further prospective studies or RCTs are warranted to comprehensively evaluate and potentially improve synthetic bone grafts.

In conclusion, synthetic bone grafts show promise in achieving comparable outcomes to autografts in radiological, clinical, and quality-of-life measures, while also minimizing complications. Procedures requiring less structural support, such as those involving cancellous bone grafts, appear to benefit most from synthetic options. However, the variability in the data collected and the limited sample size and diversity of the studies underscore the need for further research to confirm these findings with greater certainty.

### Supplementary Information

Supplementary Material 1. 

## Data Availability

The data used and/or analyzed during the current study are available from the corresponding author on reasonable request.
