# Supplementary material for: Effectiveness of synthetic versus autologous bone grafts in foot and ankle surgery: a systematic review and meta-analysis
Source: BMC Musculoskelet Disord. 2024 Jul 13;25:539. doi: 10.1186/s12891-024-07676-8 (PMC11245794; doi:10.1186/s12891-024-07676-8)
Supplement: Supplementary file 1 — Supplementary Material 1. [file 12891_2024_7676_MOESM1_ESM.docx]

| **Table S1.** Complete search strategy | |
| --- | --- |
| **Database** | **search string** |
| PubMed | (("Bone substitutes"[MeSH] OR "synthetic bone graft"[Title/Abstract] OR "bone substitute*"[Title/Abstract] OR "bone ceramic"[Title/Abstract] OR "ceramic bone"[Title/Abstract] OR "bone tissue engineering"[Title/Abstract] OR "artificial bone"[Title/Abstract] OR "calcium phosphate ceramic"[Title/Abstract] OR "tricalcium phosphate"[Title/Abstract] OR "biphasic calcium phosphate"[Title/Abstract] OR "Hydroxyapatite"[Title/Abstract] OR "calcium phosphate cements"[Title/Abstract] OR "calcium sulfate"[Title/Abstract] OR "bioactive glass"[Title/Abstract] OR "PMMA"[Title/Abstract] OR "polymethylmethacrylate"[Title/Abstract] OR "silicon*"[Title/Abstract] OR "Pyrophosphate"[Title/Abstract] OR "rhPDGF-BB"[Title/Abstract] OR "Recombinant human platelet-derived growth factor-BB"[Title/Abstract]) AND ("autogenous"[Title/Abstract] OR "autologous"[Title/Abstract] OR "Autograft"[Title/Abstract] OR "autologous bone graft"[Title/Abstract])) AND ("Foot"[MeSH] OR "Ankle"[MeSH] OR "Ankle joint"[MeSH] OR "Foot joints"[MeSH] OR "Foot"[Title/Abstract] OR "Ankle"[Title/Abstract] OR "Talus"[Title/Abstract] OR "Talar"[Title/Abstract] OR "Calcaneus"[Title/Abstract] OR "Calcaneal"[Title/Abstract] OR "Metatarsal"[Title/Abstract] OR "Metatarsus"[Title/Abstract] OR "Tarsal"[Title/Abstract] OR "Tarsus"[Title/Abstract]) |
| Scopus | (TITLE-ABS-KEY("Synthetic bone graft" OR "bone substitute" OR "bone substitutes" OR "bone ceramic" OR "ceramic bone" OR "bone tissue engineering" OR "artificial bone" OR "calcium phosphate ceramic" OR "tricalcium phosphate" OR "biphasic calcium phosphate" OR "Hydroxyapatite" OR "calcium phosphate cements" OR "calcium sulfate" OR "bioactive glass" OR "PMMA" OR "polymethylmethacrylate" OR "silicon*" OR "Pyrophosphate" OR "rhPDGF-BB" OR "Recombinant human platelet-derived growth factor-BB") AND TITLE-ABS-KEY("autogenous" OR "autologous" OR "Autograft" OR "autologous bone graft")) AND TITLE-ABS-KEY("Foot" OR "Ankle" OR "Talus" OR "Talar" OR "Calcaneus" OR "Calcaneal" OR "Metatarsal" OR "Metatarsus" OR "Tarsal" OR "Tarsus") |
| Web of Science | ((ALL=("Synthetic bone graft" OR "bone substitute" OR "bone substitutes" OR "bone ceramic" OR "ceramic bone" OR "bone tissue engineering" OR "artificial bone" OR "calcium phosphate ceramic" OR "tricalcium phosphate" OR "biphasic calcium phosphate" OR "Hydroxyapatite" OR "calcium phosphate cements" OR "calcium sulfate" OR "bioactive glass" OR "PMMA" OR "polymethylmethacrylate" OR "silicon*" OR "Pyrophosphate" OR "rhPDGF-BB" OR "Recombinant human platelet-derived growth factor-BB")) AND (ALL=("autogenous" OR "autologous" OR "Autograft" OR "autologous bone graft"))) AND (ALL=("Foot" OR "Ankle" OR "Talus" OR "Talar" OR "Calcaneus" OR "Calcaneal" OR "Metatarsal" OR "Metatarsus" OR "Tarsal" OR "Tarsus")) |
| Cochrane online library | #1 Mesh descriptor: [Bone substitutes] explode all trees  #2 "Synthetic bone graft":ti,ab,kw OR "bone substitute":ti,ab,kw OR "bone substitutes":ti,ab,kw OR "bone ceramic":ti,ab,kw OR "ceramic bone":ti,ab,kw OR "bone tissue engineering":ti,ab,kw OR "artificial bone":ti,ab,kw OR "calcium phosphate ceramic":ti,ab,kw OR "tricalcium phosphate":ti,ab,kw OR "biphasic calcium phosphate":ti,ab,kw OR "Hydroxyapatite":ti,ab,kw OR "calcium phosphate cements":ti,ab,kw OR "calcium sulfate":ti,ab,kw OR "bioactive glass":ti,ab,kw OR "PMMA":ti,ab,kw OR "polymethylmethacrylate":ti,ab,kw OR silicon*:ti,ab,kw OR "Pyrophosphate":ti,ab,kw OR "rhPDGF-BB":ti,ab,kw OR "Recombinant human platelet-derived growth factor-BB":ti,ab,kw  #3 #1 OR #2  #4 "autogenous":ti,ab,kw OR "autologous":ti,ab,kw OR "Autograft":ti,ab,kw OR "autologous bone graft":ti,ab,kw  #5 #3 AND #4  #6 Mesh descriptor: [foot] explode all trees  #7 Mesh descriptor: [ankle] explode all trees  #8 Mesh descriptor: [ankle joint] explode all trees  #9 Mesh descriptor: [food joints] explode all trees  #10 "Foot":ti,ab,kw OR "Ankle":ti,ab,kw OR "Talus":ti,ab,kw OR "Talar":ti,ab,kw OR "Calcaneus":ti,ab,kw OR "Calcaneal":ti,ab,kw OR "Metatarsal":ti,ab,kw OR "Metatarsus":ti,ab,kw OR "Tarsal":ti,ab,kw OR "Tarsus":ti,ab,kw  #11 #6 OR #7 OR #8 OR #9 OR #10  #12 #5 AND #11 |

| **Table S2.** Risk of bias Cochrane Risk of Bias Assessment Tool version 2 for randomized controlled trials | | | | | | |
| --- | --- | --- | --- | --- | --- | --- |
| **Author, year** | **Bias from randomization** | **Bias from deviations from intended interventions** | **Bias due to missing outcome data** | **Bias in measurement of the outcome** | **Bias in selection of the reported results** | **Overall risk of bias** |
| Digiovanni et al., 2013 | Low | Some | Low | Low | Low | Some |
| Daniels et al., 2019 | Low | Low | Low | Low | Low | Low |
| Digiovanni et al., 2011 | Low | Low | Low | Low | Low | Low |
| Glazebrook et al., 2013 | Low | High | Low | Low | Low | High |
| Pan et al., 2018 | Low | High | Low | Low | Low | High |

| **Table S3.** Risk of bias assessment for non-randomized studies with the ROBINS-I tool | | | | | | | | |
| --- | --- | --- | --- | --- | --- | --- | --- | --- |
| **Author, year** | **Bias due to confounding** | **Bias in selection of participants** | **Bias in classification of interventions** | **Bias due to deviations from intended interventions** | **Bias due to missing data** | **Bias in measurement of outcomes** | **Bias in selection of the reported results** | **Overall bias** |
| Fortina et al., 1998 | Low | Moderate | Moderate | Low | Moderate | Low | Low | Moderate |
| Lian et al., 2013 | Low | Moderate | Low | Low | Moderate | Low | Low | Moderate |
| Wan et al., 2020 | Low | Low | Low | Low | Low | Low | Low | Low |
